# Tic Douloureux, Seated in the Mental Branch of the Inferior Dental Nerve; Division of That Branch; Recovery

**Published:** 1853-04

**Authors:** 


### ARTICLE XII.

*Tic Douloureux, seated in the Mental Branch of the Inferior Dental Nerve; Division of that Branch; Recovery.* Under the care of Mr. FERGUSSON.

AMONG the different kinds of neuralgia which may afflict the human frame, tic douloureux, or neuralgia of the face, is the least frequently met with in our hospitals. Sciatica, however, is pretty often seen in the wards, for that affection is very nearly akin to rheumatism, and the latter often torments the poor. It may be that tic douloureux is now and then noticed in the out-patients' room, but it would appear withal that the laboring classes are not so subject to the complaint as the other portions of society. The disease is at any rate extremely difficult of cure in all, especially when it is purely neuralgic, and not sympathetic of some derangement at a greater or less distance from the nerve.

All the narcotic or stupifying drugs—as belladona, conium, hyoscyamus, opium, &c., are useful in the pure variety, but that they often fail completely is no novelty to practitioners. Aconitine has succeeded in various hands, though it now and then proves inadequate; the simple aconite ointment will sometimes answer; but recourse must now and then be had to the division of the nerve, with or without excising a portion of it. Of course such a proceeding can only be sanctioned when other means have proved fruitless, and when it is pretty certain that the disease is not, as it were, constitutional, and not depending on some distant irritation.

Mr. Fergusson has lately succeeded in freeing one of his patients from this very distressing affection, by dividing the branch of the fifth pair which was involved, and we are happy to put the case upon record. Before doing so, however, we would mention that we by far prefer the term "facial neuralgia" to "tic douloureux," which latter is by some exclusively used for neuralgia of the fifth, and by others for pain in any other nerve, or expansion of nerve. This creates much confusion, and the expression "facial neuralgia" entirely precludes any kind of obscurity.

The patient is a man about thirty-five years of age; of fair complexion, and rather thin; who was admitted under the care of Mr. Fergusson in June, 1852. He had been suffering for several years past with facial neuralgia on the right side, just at the point where the mental portion of the inferior dental nerve emerges from the mental foramen. We had observed this patient at St. George's Hospital for the last two years, where he was successively under the care of Dr. Wilson and Dr. Bence Jones. The neuralgia was at that period extremely severe; and so much so that the man was constantly rubbing the right side of his chin, the skin of which was thereby extensively ulcerated. A peculiar symptom at that period was a very profuse salivation; which latter was probably depending on irritation being conveyed from the mental branch of the inferior dental nerve, to the tonsils and salivary glands, by means of the gustatory nerve, and its tonsilitic and submaxillary branches.

The most approved remedies were used at St. George's to give the patient relief, and this end was to some degree attained; but the pain always returned. At last he applied to this hospital, and was placed under the care of Mr. Fergusson; the patient's wish being that some surgical operation should be performed, in order to free him, if possible, from the continual pain he was enduring.

The lower portion of the face on the right side was in a highly ulcerated state, from the continual rubbing which the man could not prevent himself from carrying on. Whilst Mr. Fergusson was considering the propriety of dividing the mental branch of the inferior dental nerve, the patient was attacked by fever, and was transferred to the care of Dr. Todd. It is worthy of notice, that during all the time the pyretic symptoms lasted, the facial neuralgia completely left the patient, and returned gradually as convalescence became established. When the man had quite recovered from the fever, he returned to the surgical wards, and on July 23rd, 1852, was brought into the theatre to have the above mentioned nervous branch divided.

He was narcotized by chloroform, and Mr. Fergusson, after having well ascertained the point where the mental branch of the inferior dental nerve emerges from the mental foramen in the lower jaw on the right side, made a subcutaneous section of that branch in cutting towards the bone. The part was dressed in the same manner as is usually done after tenotomy, and the patient removed. It was noticed that he evinced some symptoms of pain when he retired from the theatre.

Mr. Fergusson stated, after the operation, that the proceedings he had just adopted offered a very good prospect of relief in the present case; the section which had been made was not an infallible remedy, but it had been very effectual in several cases upon record. Mr. Fergusson thought that this measure might now be had recourse to more frequently, as with the aid of chloroform patients experience no pain, and could certainly not be made worse by the operation. When it was well ascertained which branch of the fifth pair was the seat of the neuralgia, a flap might be dissected over its course, and a piece of the nerve be excised; but whether the surgeon merely made a subcutaneous section or cut out a piece of the nerve, relief was generally obtained. Mr. Fergusson had seen, a few days previously, a gentleman upon whom he had performed this operation eighteen months before, and the pain had not as yet returned. It had been noticed, with the present patient, that he had had paroxysms of pain, even when put under the influence of chloroform; and though he seemed to suffer on leaving the table, it was very likely that in a few days the uneasiness in the nerve would have quite ceased.

This patient progressed extremely well, the wound healed rapidly, and he left the hospital a few weeks after admission, freed, at least for a time, from the very severe pain to which he had so long been subject.

This result is well calculated to revive, in some degree, this operation, which does not seem to be generally performed. With the aid of chloroform, as has been justly remarked by Mr.

Fergusson, we inflict no pain upon our patients, and it would perhaps be well that the section of some branch of the fifth pair, when the facial neuralgia is very severe, should be more frequently resorted to, especially in those cases in which the neuralgia is not sympathetic, and where the usual topical remedies have failed.

M. Jules Roux, chief surgeon of the Toulon dockyard, has lately published, in *L' Union Médicale,* an able paper on this operation, founded upon eleven cases of facial neuralgia, in which he has performed it. He thinks resection of a portion of the nerve preferable to mere division, after which the two ends unite so rapidly again, and he combines cauterization with the removal of a portion of the nerve. The author says that, at the infra-orbital foramen, almost an inch of the nerve may be taken off, and as much as one inch and a half may be removed in the inferior dental canal. He therefore prefers performing the resection of the inferior dental nerve, below and behind the last molar, to the simple section of the mental branch. M. Roux reduces from his experience that the operation is likely to be successful if the nerve is operated upon within the canal through which it courses, or at the hole from which it emerges: and in the latter case he cauterizes as high up into the canal as the caustic will reach.

To reach the inferior dental nerve in the canal, the author makes a curved incision with the concavity upwards, from the anterior margin of the masseter to the mental foramen in front of the facial artery, which latter should be compressed by an assistant. The flap is then raised, the bone laid bare, and the trephine applied. When the instrument has perforated the compact tissue of the bone, the round piece is removed with the elevator, and the nerve is easily recognized by its color, form, and direction. A grooved director is then passed under it, and a piece cut out with the scissors. The caustic is afterwards to be applied within the upper and lower portion of the dental canal, as far as the stic will go, and a great extent of the nerve is thus destroyed. The wound generally heals by first intention, and leaves a very indistinct mark.

